# Cannabidiol attenuates atherosclerosis in male ApoE^−/−^ mice with sex-dependent lipidomic and proteomic remodeling

**DOI:** 10.1016/j.jlr.2026.101120

**Published:** 2026-08-10

**Authors:** Monika Cahová, Elisabeth Ableitner, Kamila Bechyňská, Štěpánka Kučková, Jitka Viktorová, Viktória Mikušová, Vít Kosek, Zuzana Bínová, František Beneš, Ondřej Fabián, Ondřej Strnad, Tomáš Nejedlý, Marie Heczková, Miriam Brátová, Petr Kaštánek, Martin Malý, Jana Hajšlová, Marion Mussbacher, Milena Stránská

**Affiliations:** 1 Centre of Experimental Medicine, Institute for Clinical and Experimental Medicine, Prague, Czech Republic; 2 Department of Pharmacology and Toxicology, University of Graz, Graz, Austria; 3 Department of Food Analysis and Nutrition, University of Chemistry and Technology, Prague, Czech Republic; 4 Department of Biochemistry and Microbiology, University of Chemical Technology, Prague, Czech Republic; 5 Department of Data Science, Institute for Clinical and Experimental Medicine, Prague, Czech Republic; 6 Department of Pathology, Institute for Clinical and Experimental Medicine, Prague, Czech Republic; 7 Department of Biotechnology, University of Chemical Technology, Prague, Czech Republic; 8 Cardiac Centre, Institute for Clinical and Experimental Medicine, Prague, Czech Republic

**Keywords:** Cannabidiol, Atherosclerosis, Lipidomics, Proteomics, Sex differences

## Abstract

Cannabidiol (CBD), a non-psychoactive phytocannabinoid from *Cannabis sativa*, exhibits anti-inflammatory and antioxidant properties. We therefore hypothesized that CBD may modulate atherosclerosis development; however, preclinical evidence remains limited and sex-specific effects are poorly understood. Male and female apolipoprotein E-deficient (ApoE^−/−^) mice were fed a Western-type diet for 12 weeks and received either a CBD nanoemulsion (≈80 mg/kg/day) or vehicle via drinking water. Atherosclerosis was quantified by aortic plaque area, and lipidomic profiling together with aortic root proteomics were used to characterize CBD-induced metabolic changes. CBD treatment significantly reduced aortic plaque area in male but not female mice, without affecting body weight or standard serum lipid parameters. Untargeted lipidomics revealed sex-specific remodeling of the serum lipidome in males, including enrichment of ether-linked triacylglycerols, a class connected to ether-lipid metabolism; however, no lipid class emerged as a robust correlate of plaque burden. Proteomic analysis identified male-specific downregulation of mitochondrial oxidative and stress-related pathways, consistent with reduced vascular oxidative burden. *In vitro*, CBD attenuated oxLDL-induced oxidative stress and inflammatory activation in endothelial cells, supporting a direct vascular effect. CBD elicited no comparable molecular or plaque changes in females. Collectively, chronic CBD administration exerts a sex-dependent, anti-atherogenic effect in male ApoE^−/−^ mice, associated with downregulation of mitochondrial oxidative metabolism and attenuation of endothelial oxidative and inflammatory activation, alongside remodeling of ether-linked lipid metabolism whose contribution to plaque protection remains to be established. These findings highlight the importance of incorporating sex-specific responses in future mechanistic and translational studies of CBD in atherosclerosis.

Cannabinoids are bioactive compounds structurally related to metabolites of dietary essential polyunsaturated fatty acids, collectively termed endocannabinoids. They bind, with variable affinity, to a broad spectrum of endocannabinoid receptors (1), which contributes to their pleiotropic and sometimes contradictory biological effects. Cannabinoids are also important modulators of immune function (2). Given the central role of inflammation in atherogenesis (3), modulation of the endocannabinoid system has therefore been proposed as a potential strategy for atherosclerosis prevention and therapy. Early research predominantly focused on selective manipulation of the main cannabinoid receptors, either via CB1 antagonism or CB2 activation. Although these receptor-specific interventions demonstrated promising effects in preclinical models, clinical translation has been disappointing; for example, the CB1 antagonist rimonabant failed to reduce atherosclerosis progression in human trials (4) and was subsequently withdrawn due to adverse psychiatric effects (5, 6).

An alternative strategy involves naturally occurring cannabinoids with broader receptor interactions, such as cannabidiol (CBD). This non-psychoactive phytocannabinoid from *Cannabis sativa* interacts with more than 50 cardiovascular-related receptors, allosterically inhibits CB1 activation, and predominantly signals through non-canonical pathways (7). By engaging multiple molecular targets, CBD may influence several processes relevant to the pathogenesis of atherosclerosis, including oxidative stress, inflammation, and vascular remodeling. Numerous in vitro and in vivo studies have documented CBD-mediated modulation of interleukin signaling and bidirectional regulation of pro- and anti-inflammatory cytokines (8, 9, 10, 11, 12, 13), and broader immune effects have been comprehensively reviewed (14). Preclinical human studies further report CBD-induced reductions in oxidative stress and inflammation, activation of adenosine receptors, and increased angiotensin type 2 receptor expression (15). These pleiotropic activities suggest that CBD could modulate the complex pathophysiology of atherosclerosis more effectively than single-receptor interventions. However, its low oral bioavailability, rapid metabolism, and variable absorption kinetics across species remain important limitations (16), prompting interest in nanoformulations designed to enhance delivery and efficacy (17, 18).

This study addresses the largely unexplored question of whether CBD can modulate atherosclerotic plaque development. To our knowledge, this is the first investigation to evaluate chronic CBD administration in ApoE^−/−^ mice using atherosclerotic plaque burden as the primary endpoint, while simultaneously integrating aortic proteomics with serum lipidomics to provide a comprehensive characterization of pathways and mechanisms underlying CBD’s actions in the context of atherosclerosis development.

## Materials and methods

### CBD nanoemulsion

CBD nanoemulsion was prepared according to a published protocol (19) with minor modifications. The formulation consisted of 1% (*w/w*) CBD (Cerilliant Corporation), 5% (*w/w*) refined olive oil, 7% (*w/w*) emulsifier Kolliphor EL (Biomedica), and 87% (*w/w*) deionized water (dH_2_O). In the preparation process, CBD was mixed with olive oil and allowed to dissolve at room temperature with continuous stirring. Following this, an emulsifier was added to the mixture, which was then thoroughly stirred. Simultaneous homogenization was performed using an Ultra-Turrax IKA T25 homogenizer (IKA-Werke GmbH & Co. KG), during which the oil phase was incrementally added to the aqueous phase (dH_2_O). The total homogenization time from the start of the oil phase addition was recorded as 8 min at 7,500 rpm. The resulting coarse emulsion underwent further homogenization using a microfluidic homogenization technique with a Microfluidizer M-110P (Microfluidics). The emulsion was subjected to three passes through the chamber at a pressure of 2000 bar, maintaining continuous cooling throughout the process.

### Animals and study design

ApoE^−/−^ mice (B6.129P2-*Apoe^tm1Unc^*/J) of both sexes were obtained from The Jackson Laboratory at six weeks of age and randomly assigned to four groups (n = 8 per group). Upon arrival, animals were acclimatized for one week under controlled conditions while maintained on a standard chow diet for laboratory rodents (Teklad Global 18% Protein Rodent Diet, 2918; Envigo Teklad, now Inotiv), after which the experimental diet was introduced. Mice were then fed a Western-type diet (TD.88137, Envigo Teklad; now Inotiv) for 12 weeks. This purified, milk-fat-based diet provided 42% of energy from fat (21.2% fat by weight) and contained 0.2% cholesterol and 34% sucrose by weight. The control groups received 0.04% Kolliphor in drinking water, while the experimental groups received a CBD-Kolliphor nanoemulsion corresponding to ∼80 mg/kg/day. Systemic exposure to CBD was confirmed by measurable serum CBD concentrations in treated mice at the end of the experiment (M_CBD 0.77 ± 0.6 μM, F_CBD 0.72 ± 0.4 μM), while no CBD was detected in vehicle-treated controls. Body weight and water intake were monitored regularly. As expected, male mice gained more weight than females; however, CBD administration did not affect body weight gain in either sex, and water intake remained stable across all treatment groups (Supplementary Fig. S1). All procedures were approved by the Ministry of Health Ethics Committee (28132/2019-MZE-18134). A small reference cohort of C57BL/6J mice (n = 3 per sex), also obtained from The Jackson Laboratory and fed a standard chow diet, was included for baseline comparisons.

### Serum CBD quantification and clinical lipid profile

Serum samples (20 μl) were precipitated with acetonitrile (1:3, v/v), vortexed and centrifuged. The supernatant was transferred to vials for U-HPLC-HRMS/MS analysis. Targeted quantification of CBD was performed using our previously published method (19). In brief, chromatographic separation was performed on a C18 reversed-phase column (Acquity UPLC BEH C18 (150 × 2.1 mm; 1.7 μm, Waters) using a water–acetonitrile gradient with ammonium formate and formic acid. Detection was carried out on a high-resolution Q-Orbitrap mass spectrometer operated in ESI(+), using full MS (200–600 m/z) and a resolving power of 35,000 full width at half maximum (FWHM). Quantification relied on external calibration with matrix-matched standards; an isotopically labeled internal standard (CBD-D_3,_ Cerilliant Corporation) was added to all samples and calibration points.

### Lipidomic profiling

Serum samples (20 μl) were spiked with 20 μl of Mouse SPLASH® LIPIDOMIX® Mass Spec Standard (Avanti Polar Lipids) and extracted using MTBE/MeOH liquid–liquid extraction. After centrifugation, 180 μl of the upper organic phase was transferred to a microtube, evaporated to dryness, and reconstituted in 180 μl of iPrOH:MeOH:H_2_O (65:30:5, *v:v:v*) prior to analysis. Pooled quality control (QC) samples (group-specific pools and an overall pooled QC) were prepared to monitor instrument stability and to acquire MS/MS spectra for lipid annotation.

For the U-HPLC-HRMS/MS analysis, U-HPLC Vanquish Horizon (Thermo Fisher Scientific) coupled to a high-resolution tandem mass spectrometer Exploris 240 (Thermo Fisher Scientific) with the heated electrospray (H-ESI) source were employed. For chromatographic separation of sample components, Acquity BEH C18 (2.1 mm × 150 mm; 1.7 μm (Waters)) was used. The chromatographic system used with ESI+ detection was: A – 10 mM ammonium formate and 0.1% formic acid in ACN:dH_2_O (60:40, *v:v*); B – 10 mM ammonium formate and 0.1% formic acid in iPrOH:ACN (90:10, *v:v*). For chromatographic separation of lipids detected in ESI- mode, the following mobile phases were used: A – 10 mM ammonium acetate and 0.1% acetic acid in ACN:dH_2_O (60:40, *v:v*); B – 10 mM ammonium acetate and 0.1% acetic acid in iPrOH:ACN (90:10, *v:v*). The flow rate was constant at 0.300 ml/min. The column temperature was maintained at 60°C, the injection volume was increased to 3 μl. The autosampler was kept at 10°C. The total run time was 21 min for ESI+ and 18 min for ESI-. The mass spectrometer was operated under the following conditions in both ionization modes: Spray voltage: Static, Ion voltage: 4,000 V, Gas mode: Static, Sheath gas: 45 Arb. Aux gas: 10 Arb, Sweep gas: 0. Ion Transfer Tube temp: 250°C, Vaporizer temp: 250°C, Orbitrap resolution: 240,000. For all samples, the data were acquired over the *m/z* range of 100-1,200 in Full Scan mode. To obtain the fragmentation spectra of lipids, the group pool samples were run in data dependant acquisition (DDA) mode with HCD collision energies 10, 20 and 40 eV, with enabled dynamic exclusion. All mass spectrometry data were processed in two consecutive steps using MZmine 2 (20). Each analytical batch (serum ESI+ and ESI−) was processed separately. For initial feature detection, all group pool samples were used together with a limited number of representative individual samples from each class to ensure coverage of both high-abundance and class-specific features. Feature detection based on exact mass was followed by chromatogram deconvolution using the Local Minimum Search algorithm. Features were aligned using an m/z tolerance of 5 ppm and a retention time tolerance of 0.1 min. Isotope peak grouping and join alignment were subsequently performed. The resulting feature list was subsequently used for targeted feature extraction. In the second step, all samples were reprocessed using targeted feature extraction based on this feature list. Lipid annotation was performed using the LipidMatch R script (21), which relies on in silico fragmentation databases. Lipid identification required the presence of at least class-specific fragment ions. When additional diagnostic fragments were available, lipid species were further characterized based on their fatty acyl composition (i.e., number of carbon atoms and double bonds in the acyl chains). All lipid annotations were supported by MS/MS spectra obtained from group-specific QC samples using repeated fragmentation at multiple collision energies. Finally, redundant features corresponding to multiple adducts of the same lipid species were manually curated, and only the adduct with the highest intensity was retained for further analysis. Lipid abundances were subsequently normalized to the corresponding class-specific, adduct-matched internal standards prior to statistical analysis.

### Aortic plaque quantification

The heart was perfused with phosphate-buffered saline via cardiac puncture to remove residual blood. The entire aorta was subsequently dissected, cleaned, and fixed overnight in 4% paraformaldehyde. Aortas were then opened longitudinally, stained with Oil Red O, and pinned en face for plaque visualization. Images were processed to remove pins using Affinity Photo software, and plaque area was quantified as the percentage of total aortic surface using ImageJ (22). All analyses were performed according to standardized protocols by two investigators blinded to group allocation. Unprocessed images are provided in Supplementary Fig. S2.

### Aortic root proteomics

The aortic root was collected for proteomic analysis. Sample preparation was performed according to a previously published protocol (23) with minor modifications. Briefly, aortic roots (∼65 mg) were cryogenically disrupted using liquid nitrogen, and proteins were sequentially extracted to obtain four fractions (FR1–FR3 and an insoluble fraction). Tissue was first homogenized in PBS-containing protease inhibitors, followed by centrifugation (20 min, 16,000 × g, 4°C). The supernatant was collected, and the pellet was subsequently extracted using buffers of increasing stringency containing Tris-HCl, NaCl, glycerol, IGEPAL CA-630, sodium deoxycholate, SDS, benzonase, and protease and phosphatase inhibitors. Each extraction step was followed by incubation and centrifugation, and the resulting supernatants (FR1–FR3) were collected. The final insoluble pellet was resuspended in PBS with protease inhibitors. All fractions were precipitated with 80% acetone and resuspended in denaturation/reduction/alkylation buffer containing guanidinium hydrochloride, Tris-HCl (pH 8), TCEP, and chloroacetamide. Samples were heat-denatured (99°C, 10 min) and sonicated, followed by overnight digestion at 37°C using trypsin and LysC. The insoluble fraction was additionally processed by urea/thiourea-assisted solubilization prior to digestion. Peptides from all fractions were desalted using reverse-phase C18 solid-phase extraction.

Peptide separation was performed by nano–liquid chromatography using a nanoElute 2 system (Bruker Daltonics). Samples were resuspended in 3% acetonitrile/0.1% formic acid and loaded onto a PepMap Neo-Trap column (300 μm × 5 mm, 5 μm particle size) at 85 bar for 2.5 min using 100% mobile phase A (0.1% formic acid in water). Peptides were then separated on a PepSep C18 analytical column (75 μm × 100 mm; 1.9 μm) using a linear gradient of 3–35% mobile phase B (0.1% formic acid in acetonitrile) over 60 min, followed by column washing at 95% B.

Eluted peptides were introduced directly into a timsTOF HT mass spectrometer (Bruker Daltonics) via a CaptiveSpray ion source operated at 1,600 V, 150°C, and a nitrogen flow of 3 L/min. Data were acquired in DIA-PASEF (Data-Independent Acquisition–Parallel Accumulation Serial Fragmentation) mode over a mass range of 100–1700 m/z with ion mobility separation (0.6–1.6 V s cm^−2^). Precursors were selected in the range of 400–1201 m/z using 32 isolation windows of 26 Da width. Only fractions FR1 and FR3 were included in the final analysis, as fractions FR2 and the insoluble fraction yielded insufficient proteome coverage and did not meet quality criteria for reliable quantification. All analyses were performed using standardized label-free quantification workflows.

### In vitro experiments

#### Cells

Human coronary artery endothelial cells (HCAEC, P10460; Innoprot) were used in all in vitro tests. According to the manufacturer’s recommendations, the cells can be further cultured for up to 10 passages; therefore, one donor cell lot was used for the experiment. In all experiments, a vehicle control (0.5% DMSO) was used. All in vitro experiments were performed in at least three independent biological replicates.

#### Cell viability assay

Viability of HCAEC was assessed using a resazurin-based assay. Cells were seeded into 96-well plates at a density of 5 × 10^4^ cells per well and allowed to adhere overnight. The following day, the medium was replaced with fresh endothelial growth medium (Endothelial Basal Medium, Innoprot) supplemented with 5% fetal bovine serum, endothelial cell growth supplement, penicillin/streptomycin, and increasing concentrations of cannabidiol (CBD; Cerilliant Corporation; 0.6–20 mg/L). The final concentration of DMSO did not exceed 0.5%. Cells were incubated for 72 h at 37°C in a humidified atmosphere with 5% CO_2_. Subsequently, the medium was replaced with 100 μl of resazurin solution (0.03 g/L in PBS), and cells were incubated for an additional 1 h. Fluorescence was measured at excitation/emission wavelengths of 560/590 nm using a SpectraMax i3x Multi-Mode Microplate Reader (Molecular Devices, USA) to assess metabolic activity. Cell viability was expressed as a percentage of untreated controls, and the half-maximal inhibitory concentration (IC_50_) of CBD was determined from dose–response curves using nonlinear regression analysis in GraphPad Prism (version 7).

#### Cytokine quantification

Inflammatory activation of human coronary artery endothelial cells (HCAEC) was assessed by measuring cytokine production using a sandwich enzyme-linked immunosorbent assay (ELISA). Cells were seeded into 96-well plates at a density of 5 × 10^4^ cells per well and allowed to adhere overnight. The following day, the medium was replaced with endothelial basal medium supplemented with 0.5% fetal bovine serum, 0.3% fatty acid-free bovine serum albumin, and endothelial cell growth supplement. Cells were treated with oxidized low-density lipoprotein from human plasma (oxLDL, 50 mg/L; L34357, Thermo Fisher Scientific) in the presence or absence of cannabidiol (CBD; 2.5 mg/L), as indicated. The final concentration of DMSO did not exceed 0.5%. After 24 h of incubation, culture supernatants were collected and centrifuged (1000 × g, 15 min, 4°C) to remove cellular debris. Concentrations of interleukin-6 (IL-6) and monocyte chemoattractant protein-1 (MCP-1) were quantified using sandwich ELISA. Briefly, 96-well plates were coated with cytokine-specific capture antibodies, blocked with casein, and incubated with samples, followed by biotinylated detection antibodies and HRP-conjugated avidin. Signal was developed using a chromogenic HRP substrate, and absorbance was measured at 450 nm using a microplate reader.

#### Western blot analysis

Protein expression of intercellular adhesion molecule-1 (ICAM-1) in human coronary artery endothelial cells (HCAEC) was analysed by Western blotting. Cells were seeded onto 60 mm culture dishes at a density of 5 × 10^4^ cells/ml and allowed to adhere overnight. Subsequently, cells were treated with oxidized low-density lipoprotein (oxLDL; 50 mg/L) and lipopolysaccharides (LPS, 0.1 mg/L; L4516, Merck) in the presence or absence of cannabidiol (CBD; 2.5 mg/L) for 24 h. The final concentration of DMSO did not exceed 0.5%.

Following treatment, cells were washed with PBS and lysed in phosphate-based lysis buffer containing sodium deoxycholate and Triton X-100. Protein concentration was determined using a BCA assay. Equal amounts of total protein (2 μg) were separated by SDS–PAGE on 10% polyacrylamide gels and transferred onto nitrocellulose membranes. Membranes were blocked with 5% non-fat dry milk in PBST and incubated with a primary antibody against ICAM-1 (PA5-82002, Thermo Fisher Scientific, USA). After washing, membranes were incubated with HRP-conjugated secondary antibodies, and protein bands were visualized using a chemiluminescent substrate (Immobilon Forte Western HRP substrate, Merck). β-actin served as a loading control.

#### Cellular antioxidant activity assay

Cellular antioxidant activity in HCAEC was evaluated using the DCFH-DA assay. Cells were seeded into 96-well plates at a density of 5 × 10^4^ cells per well and allowed to adhere overnight. The following day, cells were washed with PBS and incubated for 1 h at 37°C in a humidified atmosphere with 5% CO_2_ in vascular cell basal medium containing 2′,7′-dichlorofluorescin diacetate (DCFH-DA, 0.0125 g/L; Merck). Subsequently, the medium was replaced with vascular cell basal medium supplemented with 0.5% fetal bovine serum, 0.3% bovine serum albumin, and endothelial cell growth supplement. Cells were treated with oxLDL; 50 mg/L in the presence or absence of cannabidiol (CBD; 0.6–2.5 mg/L) or ascorbic acid (0.5 g/L) as a reference antioxidant. Intracellular oxidative stress was assessed by measuring fluorescence at excitation/emission wavelengths of 485/535 nm immediately after treatment and after 2, 4, and 24 h using a microplate reader.

### Liver histology

Samples were fixed in buffered 10% formalin for at least 24 h. After that, the material was embedded in paraffin, and 3-μm histological sections were prepared and stained with hematoxylin and eosin.

### Statistical analysis

Statistical analyses were performed in R (v4.5.0) and MetaboAnalyst.

#### Clinical, hepatocellular, and plaque parameters

Normality was tested using the Shapiro–Wilk test. As none of the variables met normality, two-factor Aligned Rank Transform (ART) ANOVA was applied with Sex and Treatment as fixed effects and their interaction (ARTool package). Post hoc comparisons were performed using ART-C contrasts (art.con, ARTool package).

#### Lipidomics

Data preprocessing included log transformation and autoscaling. PCA was used for dimensionality reduction and to obtain an initial overview of the data structure. Welch's *t* test with false discovery rate (FDR) correction and fold change analysis were performed. Lipid species associated with plaque burden were initially screened using Pearson correlation (exploratory analysis; |r| ≥ 0.6). To evaluate the ether-TAG hypothesis while avoiding circularity, class-level composite indices were constructed as the mean of per-species z-scores (plaque-blind), including only lipid species that passed quality-control criteria in pooled QC samples (RSD ≤ 30%). Associations between the ether-TAG index and plaque area were assessed using robust regression (lmrob; primary analysis), with rank-based regression (rfit) and Spearman correlation performed as sensitivity analyses. Class specificity was evaluated in a plaque- and treatment-blind whole-class screen using Benjamini–Hochberg FDR correction across lipid classes. Exploratory mediation analysis (CBD → ether-TAG index → plaque area) was performed in male mice using the mediation package.

#### Proteomics

PCA was employed to visualize the data based on the first three principal components (stats v3.6.2). Permutational Multivariate Analysis of Variance (PERMANOVA) was conducted as an initial exploratory analysis to evaluate whether overall protein profiles differed between the two groups (vegan v2.6-4). Differential abundance analysis of individual proteins was performed using two complementary approaches: ROTS (Reproducibility-Optimized Test Statistic), which applies reproducibility-optimized statistics through resampling (ROTS v1.0.0), and SAM (Significance Analysis of Microarrays), which relies on permutation-based statistics (samr v3.0). To investigate the biological processes distinguishing the untreated and CBD-treated groups, a topology-based pathway analysis method, Pathway Express, on the protein fold changes from SAM, was performed in combination with the Reactome database (ROntoTools v2.18.0, ReactomePA v1.16.2).

## Results

### CBD reduces atherosclerotic plaque formation in males but not females

**Fig. 1:**
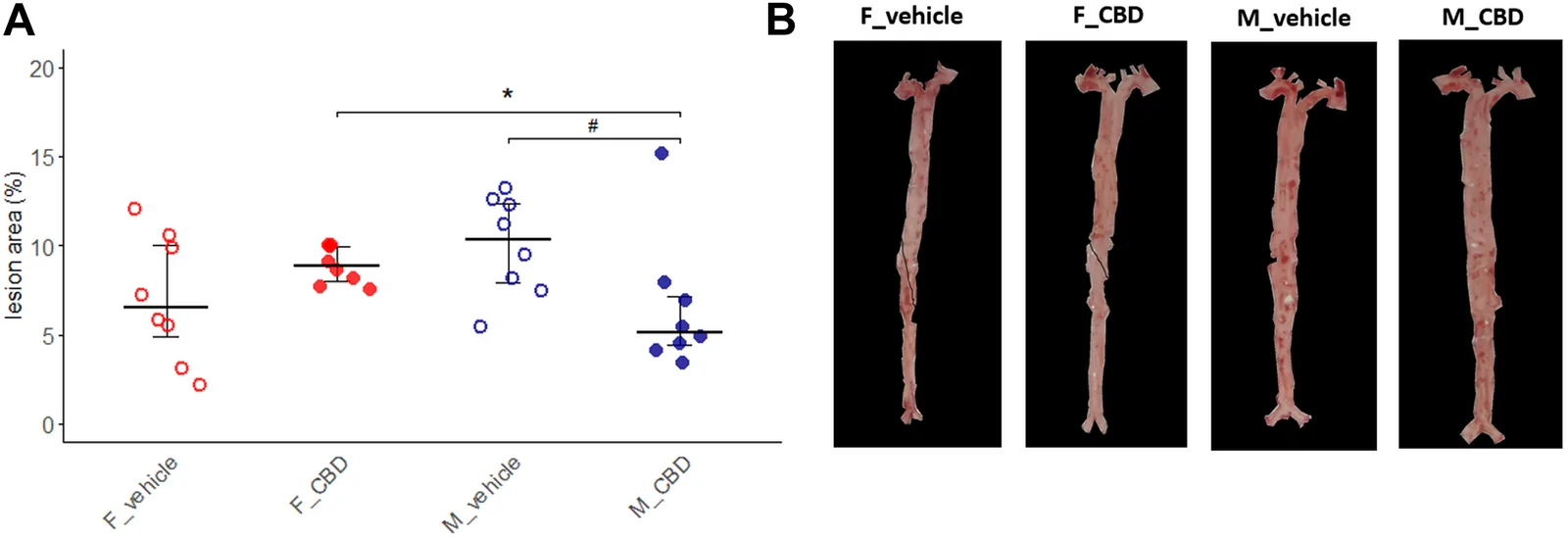
Aortic lesions and plaques in CBD-treated ApoE^−/−^ mice. A: En face analysis of oil red-stained aortas in the percentage of lesion area. Data are given as median and IQR. Each dot represents an individual mouse; black lines indicate medians. Statistical evaluation was performed by ART ANOVA with ART-C contrasts and Benjamini-Hochberg correction. B: Representative pictures of the dissected and stained aortas. M, male; F, female. #*P* < 0.05 M_vehicle versus M_CBD; ∗*P* < 0.05 F_CBD versus M_CBD.

The impact of CBD on atherosclerotic burden was assessed by *en face* quantification of aortic plaque area. ART ANOVA revealed a significant Sex × Treatment interaction (*P* = 0.009), demonstrating that CBD exerted differential effects in male and female mice. Neither sex nor treatment showed significant main effects (*P* = 0.83 and *P* = 0.368, respectively). Post hoc ART-C contrasts demonstrated a significant reduction in plaque area in CBD-treated males but not females, which was further supported by Hodges–Lehmann estimates and Wilcoxon tests (Table 1). The interaction was reproduced by both robust and rank-based factorial regression, and the male treatment effect remained significant after exclusion of a single influential animal (Supplementary Table S1). Individual values are shown in Figure 1.

**Table 1:** CBD Effect on Plaque Area Within Each Sex

Sex	Vehicle Median (%)	CBD Median (%)	HL Shift (95% CI)	Wilcoxon p	ART-C p (BH)
Male	10.35	5.13	−4.25 (−7.70, −0.20)	0.031	0.021
Female	6.5	8.85	+2.18 (−2.00, 5.40)	0.27	0.175

ART-C contrasts (post hoc to ART; BH-corrected across the two within-sex contrasts) with Hodges-Lehmann (HL) shift and Wilcoxon rank-sum test. Plaque area as percent of aortic surface.

### CBD modulates the aortic proteome in males but not females

**Fig. 2:**
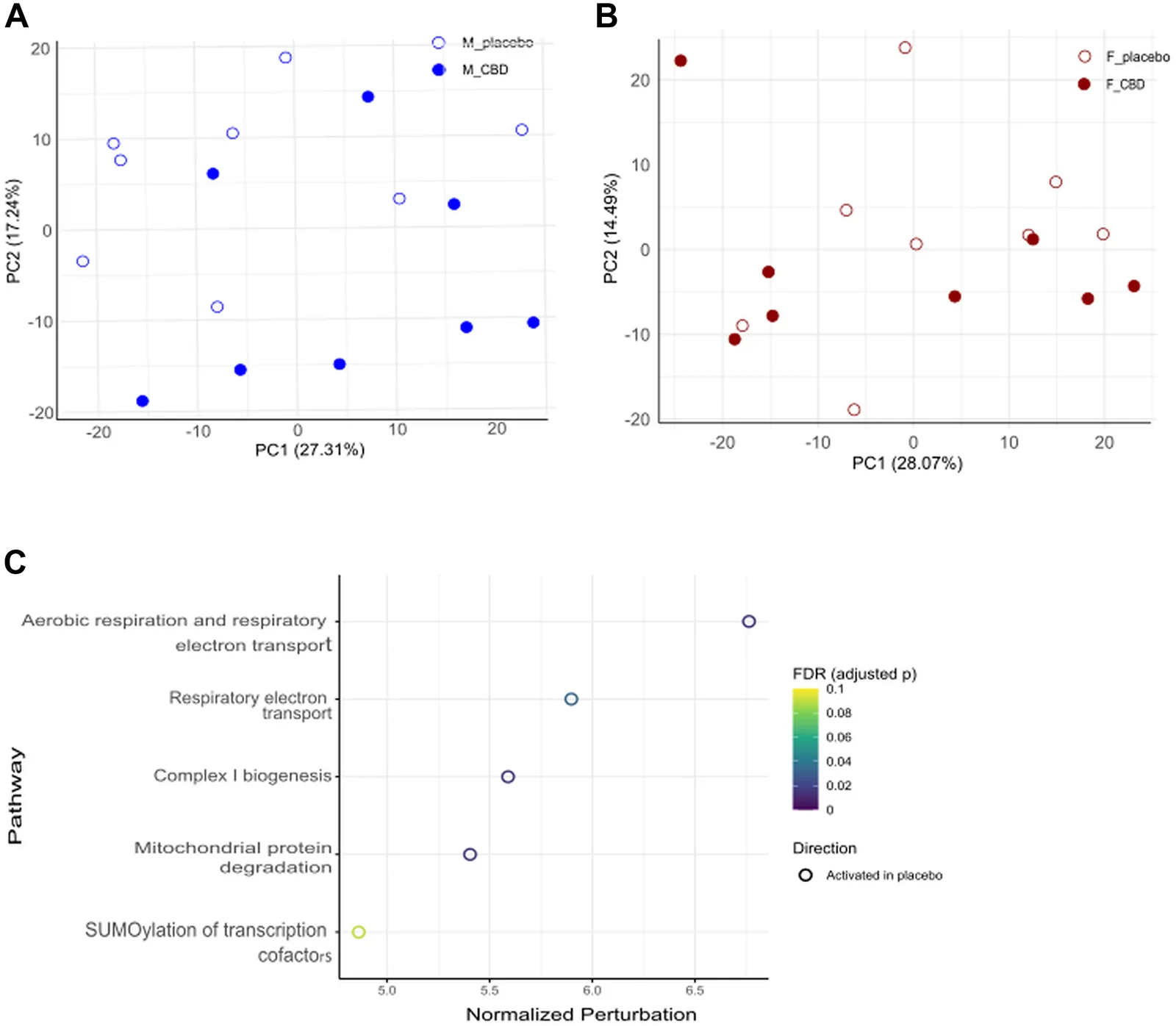
Aortic root proteome of CBD- and vehicle-treated ApoE^−/−^ mice. A, B: Principal Component Analysis of the aortic root proteome composition in CBD- and vehicle-treated groups. (A) Male mice; (B) Female mice. Each point represents one sample, with red points indicating CBD-treated animals and green points indicating vehicle-treated controls. The percent of the explained variance for each component is given in parentheses. C: Biological pathways enriched in the aortic tissue of CBD- and vehicle-treated male mice. The dot plot presents significantly enriched pathways based on the Reactome database. Each dot represents a pathway; dot size reflects the number of genes contributing to the enrichment, and color indicates the adjusted *P*-value (with darker shades representing higher statistical significance).

Proteomic profiling of the aortic root revealed sex-dependent effects of CBD (Fig. 2A, B). In males, PCA showed partial separation between the CBD and untreated groups, explaining 58.4% of variance. This separation was statistically supported by PERMANOVA (*P* = 0.048). No clustering was observed in females (*P* = 0.372), suggesting the absence of major CBD-related effects on the aortic root proteome in this sex.

Although the multivariate treatment effect in males was modest at the level of global proteome structure, it became much more apparent at the level of individual proteins. To pinpoint these specific alterations, we applied two complementary statistical methods: Reproducibility Optimized Test Statistic (ROTS), a conservative reproducibility-optimized test, and Significance Analysis of Microarrays (SAM), a more permissive permutation-based approach. ROTS identified six differentially abundant proteins (five downregulated and one upregulated) in CBD-treated males compared to controls. SAM revealed a broader set of 108 proteins, all of which were significantly less abundant under CBD treatment (FDR < 0.1) (Supplementary Fig. S3, Supplementary Table S2). These male-specific proteomic changes are consistent with the reduction of aortic plaque burden observed in CBD-treated males, suggesting that CBD may modulate atherogenesis through the deregulation of specific protein networks.

Pathway Express analysis revealed significant downregulation of mitochondrial pathways (Fig. 2C), including aerobic respiration, respiratory electron transport, complex I assembly, and mitochondrial protein degradation, in CBD-treated males (FDR < 0.1). No pathways were significantly affected in females (all FDR > 0.25).

### CBD does not alter routine serum lipids or the global structure of the lipidome

**Fig. 3:**
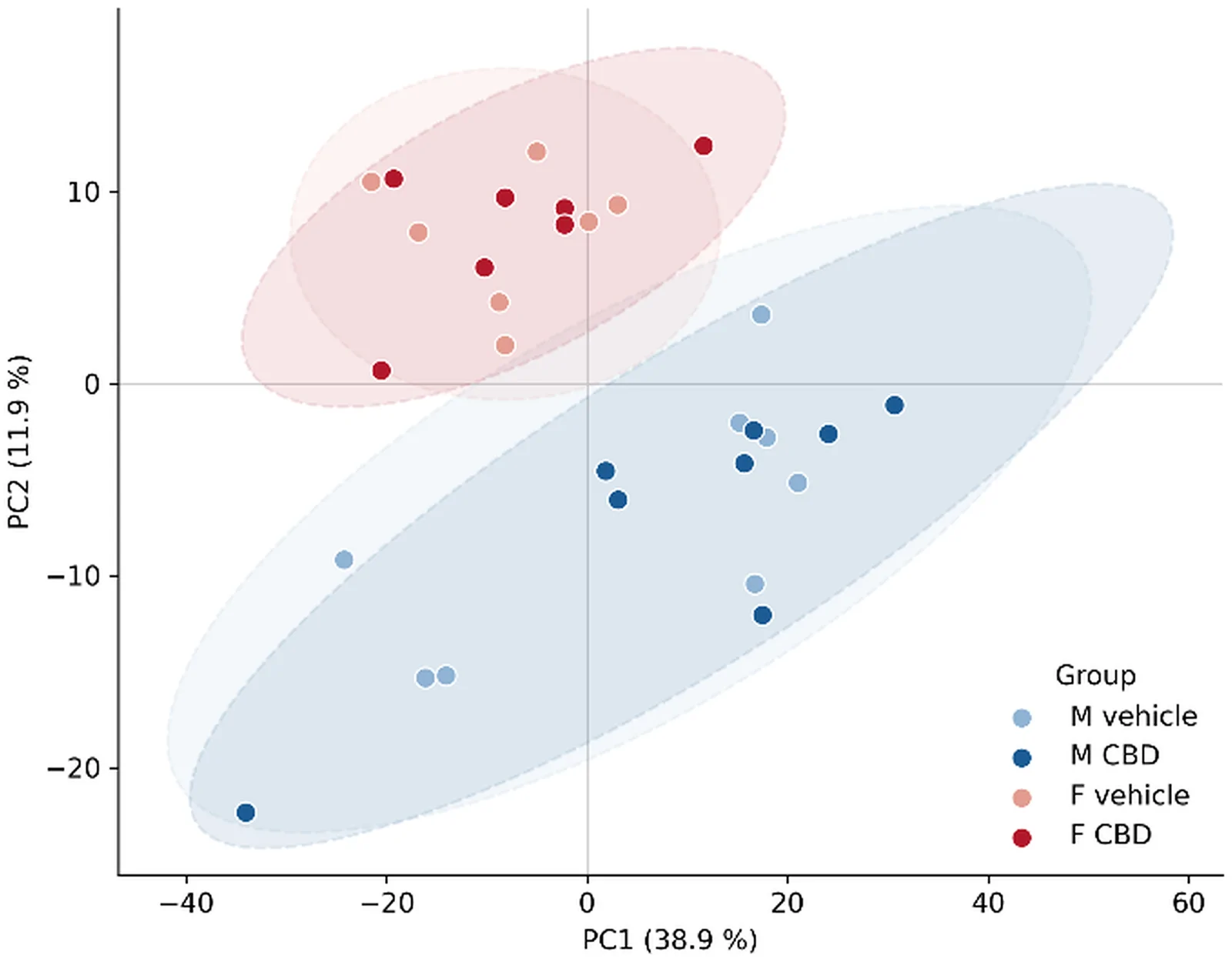
PCA of the serum lipidome. PCA was performed on all 724 lipid species without feature preselection; concentrations were log2-transformed and autoscaled. Points are individual animals (n = 30), coloured by group; shaded areas are 95% confidence ellipses. PC1 and PC2 explain 38.9% and 11.9% of the variance. Samples separate by sex, not by treatment (PERMANOVA Sex *P* = 0.0006; Treatment *P* = 0.73).

Routine serum lipid parameters (total cholesterol, HDL, LDL, and triglycerides) were compared across groups by ART ANOVA with ART-C contrasts. None of these parameters differed significantly between CBD- and vehicle-treated animals in either sex (Supplementary Table S3). To assess the circulating lipidome more broadly, we performed lipidomic profiling and identified 724 lipid species. Unsupervised principal component analysis separated samples primarily by sex, with no clear separation by treatment (Fig. 3). This pattern was confirmed by PERMANOVA, which identified a significant effect of sex (*P* = 0.0006) but no effect of treatment (*P* = 0.73). CBD therefore did not reshape the global structure of the serum lipidome, and any treatment-related effects were confined to individual lipid species rather than to the lipidome as a whole.

### In males, ether-linked triacylglycerols are the leading correlates of plaque burden

Given the absence of a global treatment effect, we asked whether individual lipid species tracked atherosclerotic burden. Within each sex, log2 abundance of every species was correlated with plaque area using Pearson and Spearman coefficients with Benjamini-Hochberg correction across the lipidome, and the CBD-versus-vehicle fold change was used to establish the direction of any treatment effect (Supplementary Tables S4 and S5).

**Fig. 4:**
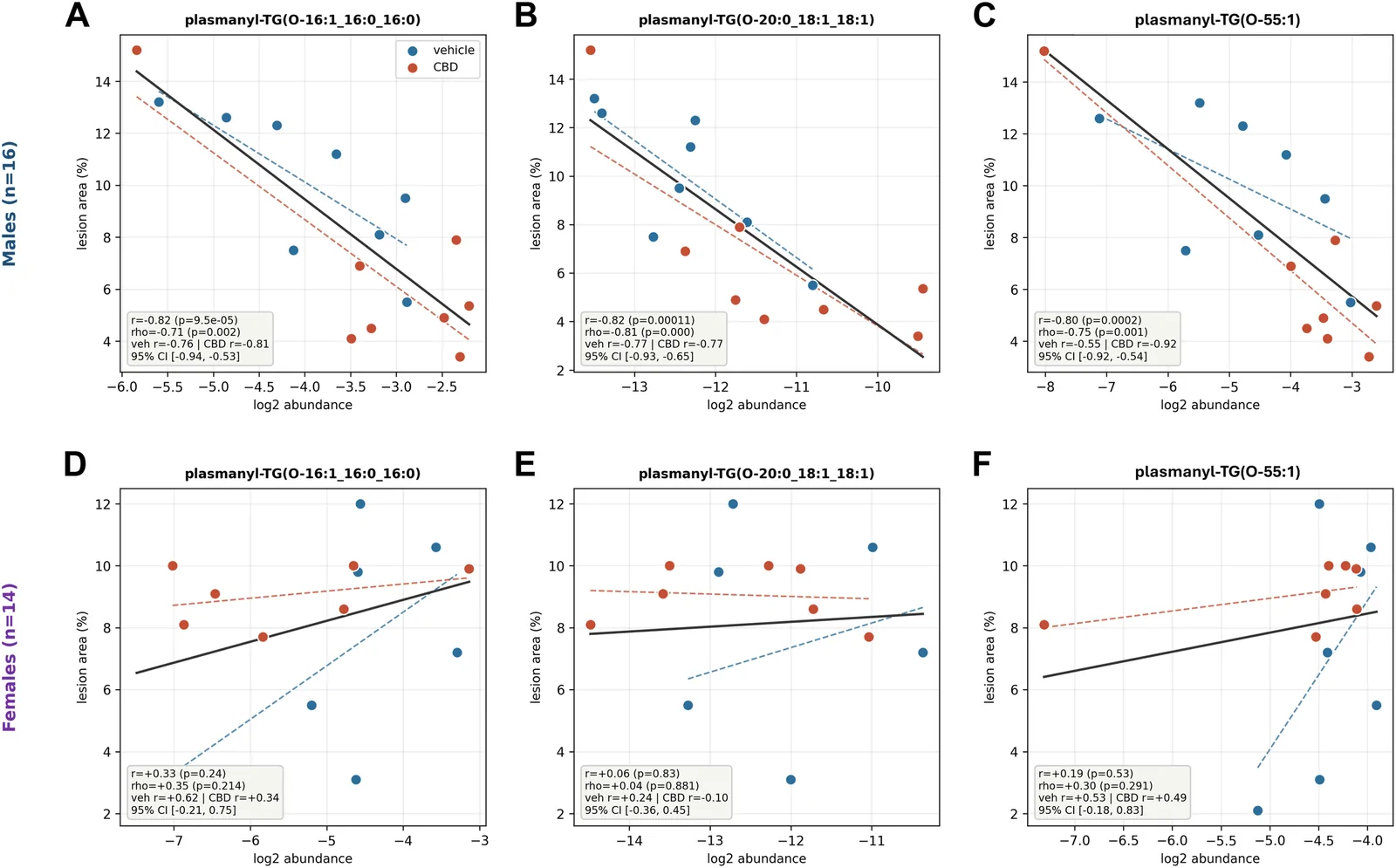
Association of representative ether-linked triacylglycerol species with aortic plaque area in male and female ApoE−/− mice. Scatterplots of aortic lesion area (%) against log2-transformed abundance of three leading plasmanyl-TAG species in males (A–C) and females (D–F). Scatterplots of aortic lesion area (%) against log2-transformed abundance of the three leading plasmanyl-TAG species in male ApoE^−/−^ mice. Points are colored by treatment (vehicle, blue; CBD, red). The solid black line represents the pooled regression across all males; dashed lines represent the within-group regressions (vehicle, *blue*; CBD, *red*). The most influential animal in each panel, identified by leave-one-out diagnostics, is marked with a black circle. Insets report the pooled Pearson correlation (log2 abundance), the Spearman rank correlation, the within-group Pearson coefficients, and the bootstrap 95% confidence interval (5,000 resamples) for the pooled correlation.

In males, the strongest correlates of plaque area formed a coherent set of negatively associated species dominated by ether-linked (plasmanyl) triacylglycerols, accompanied by selected HexCer, SM, LPC, and LPE species (Supplementary Table S4). For the leading ether-TAG species, correlations were strong and negative (Spearman rho approximately −0.7 to −0.8) and nominally significant throughout; on parametric (log2 Pearson) testing, they survived FDR correction across the lipidome, although the more conservative rank-based test left them just above the FDR threshold. Importantly, the associations were present within both the vehicle and CBD groups (Fig. 4), indicating that they were not an artifact of treatment-induced separation. Representative MS/MS spectra of the three plasmanyl-TAG species highlighted in Figure 4 are provided in Supplementary Figu. S4.

These same ether-TAG species were increased by CBD (fold change greater than one), so that higher abundance co-occurred with both CBD treatment and smaller plaques, a directionally coherent pattern consistent with a protective role. In females, no coherent association was found: the strongest correlates were weak or positive, none survived FDR correction, and CBD did not appreciably change their abundance (Supplementary Table S5). The lipid correlates of plaque burden were therefore male-specific.

### The ether-TAG association is nominal and not specific at the class level

**Fig. 5:**
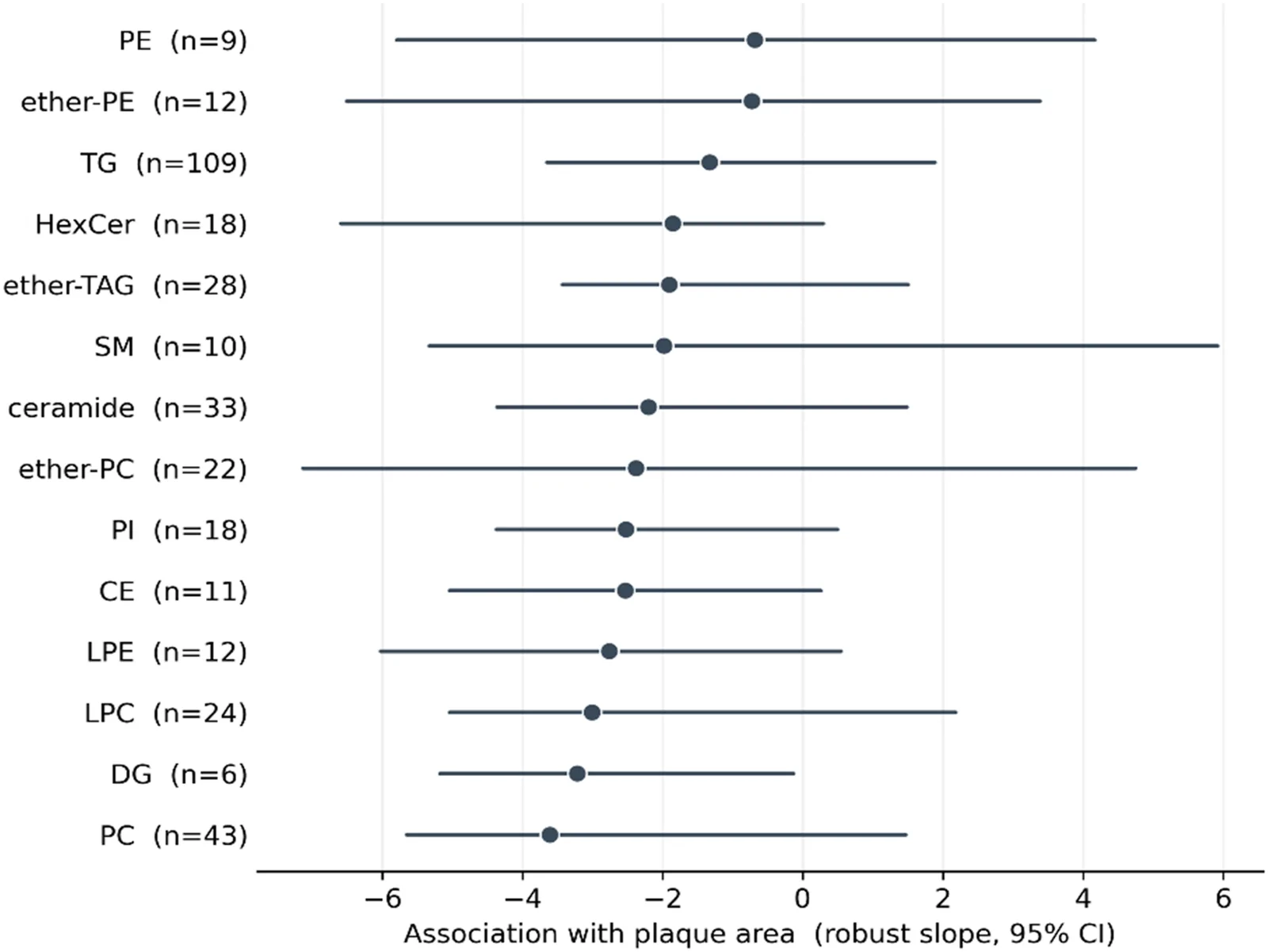
Class-level specificity screen of lipid associations with plaque burden in males. Each of the 14 detected lipid classes was condensed into a plaque-blind composite index (mean of per-species z-scores). Points show the slope of a robust regression of plaque area on the class composite in male ApoE^−/−^ mice (n = 16); horizontal lines are bootstrap 95% confidence intervals. Classes are ordered by slope; the dashed vertical line marks a slope of zero.

To test whether ether-TAG abundance tracks plaque burden beyond the selection of individual species, each lipid class was condensed into a plaque-blind composite index (mean of per-species z-scores), and plaque area was regressed on the index using robust and rank-based methods. In males, the ether-TAG index was negatively associated with plaque area, but the association was only nominal (Supplementary Table S6). When the same approach was applied class by class in a plaque- and treatment-blind manner, composites of all lipid classes trended negatively with plaque and none survived FDR correction, with the ether-TAG composite ranking in the middle of the distribution (Fig. 5). An exploratory mediation analysis of the CBD to ether-TAG to plaque path did not reach significance.

Taken together, these analyses indicate that the inverse relationship between lipid abundance and plaque burden in males is a general feature of the male lipidome rather than a property unique to ether-TAG. At the same time, ether-linked triacylglycerols were consistently the most strongly and coherently associated class, were the lipids most clearly increased by CBD, and showed a biologically plausible directionality. We therefore regard the ether-TAG signal as an exploratory but internally consistent observation that may reflect a genuine, if modest, link to plaque size.

### CBD attenuates oxLDL-induced inflammation in vitro

**Fig. 6:**
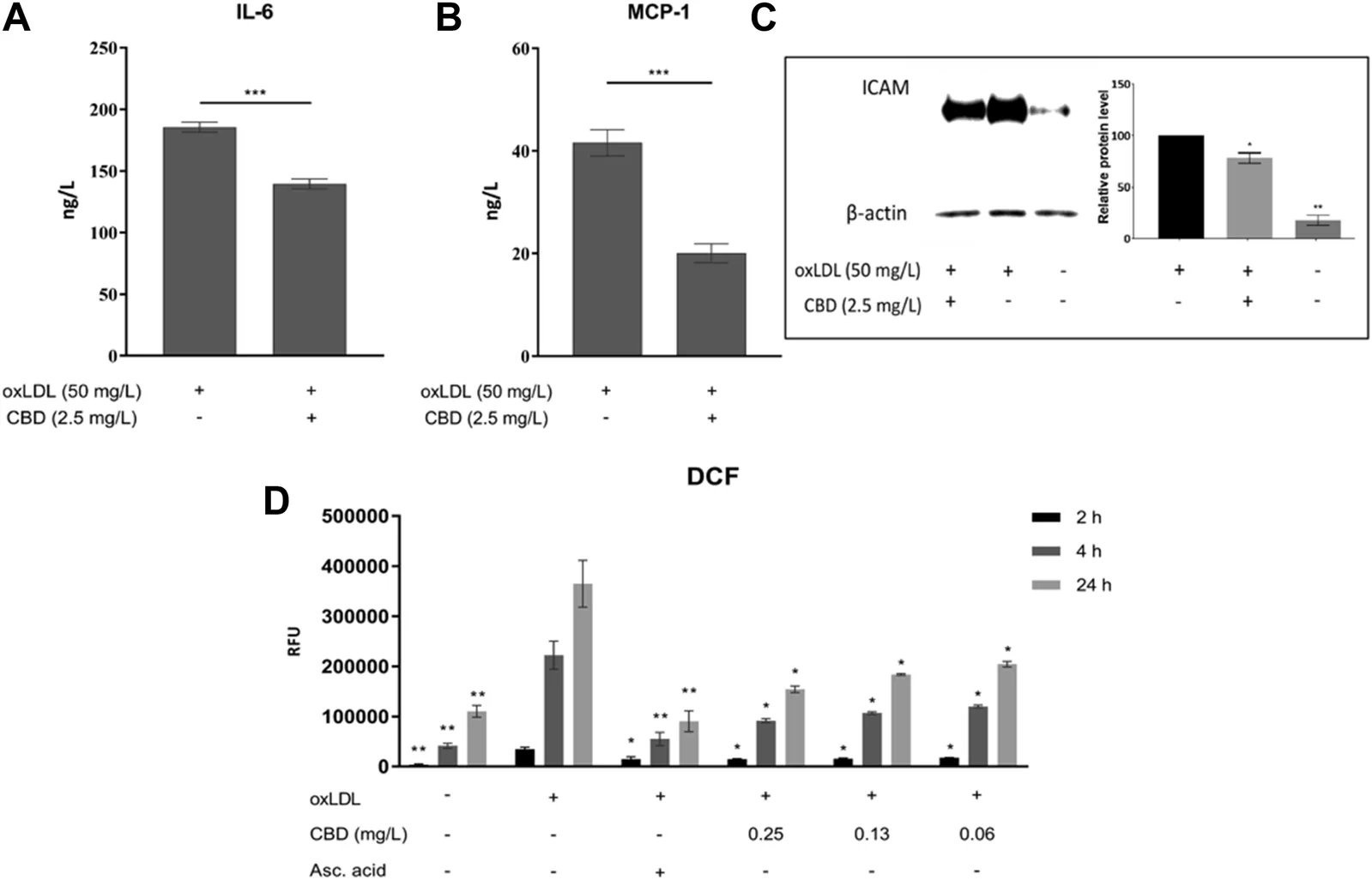
In vitro effects of CBD on oxLDL-induced endothelial activation. A and B: Effect of cannabidiol (CBD, 2.5 mg/L) on oxLDL-induced secretion of IL-6 (A) and MCP-1 (B) in human coronary artery endothelial cells (HCAEC), quantified by ELISA. Data are mean ± SEM (n = 3–4); ∗∗∗*P* < 0.001 versus oxLDL. C: Representative Western blot and densitometric quantification of ICAM protein expression in oxLDL-stimulated HCAEC treated with CBD (2.5 mg/L); β-actin served as loading control. Data are mean ± SEM (n = 3); ∗*P* < 0.05, ∗∗*P* < 0.005 versus oxLDL. All Western blot membranes are included in the supplementary materials (Supplementary Figs. S5–S7). D: Cellular antioxidant activity in oxLDL-treated HCAEC exposed to CBD (0.25, 0.13, 0.06 mg/L) or ascorbic acid (0.5 g/L). Oxidative stress was assessed by DCF fluorescence at 2, 4, and 24 h. Data are mean ± SEM (n = 4–5); ∗*P* < 0.05, ∗∗*P* < 0.01 versus oxLDL at the respective time point.

**Supplemental Figure S5:**
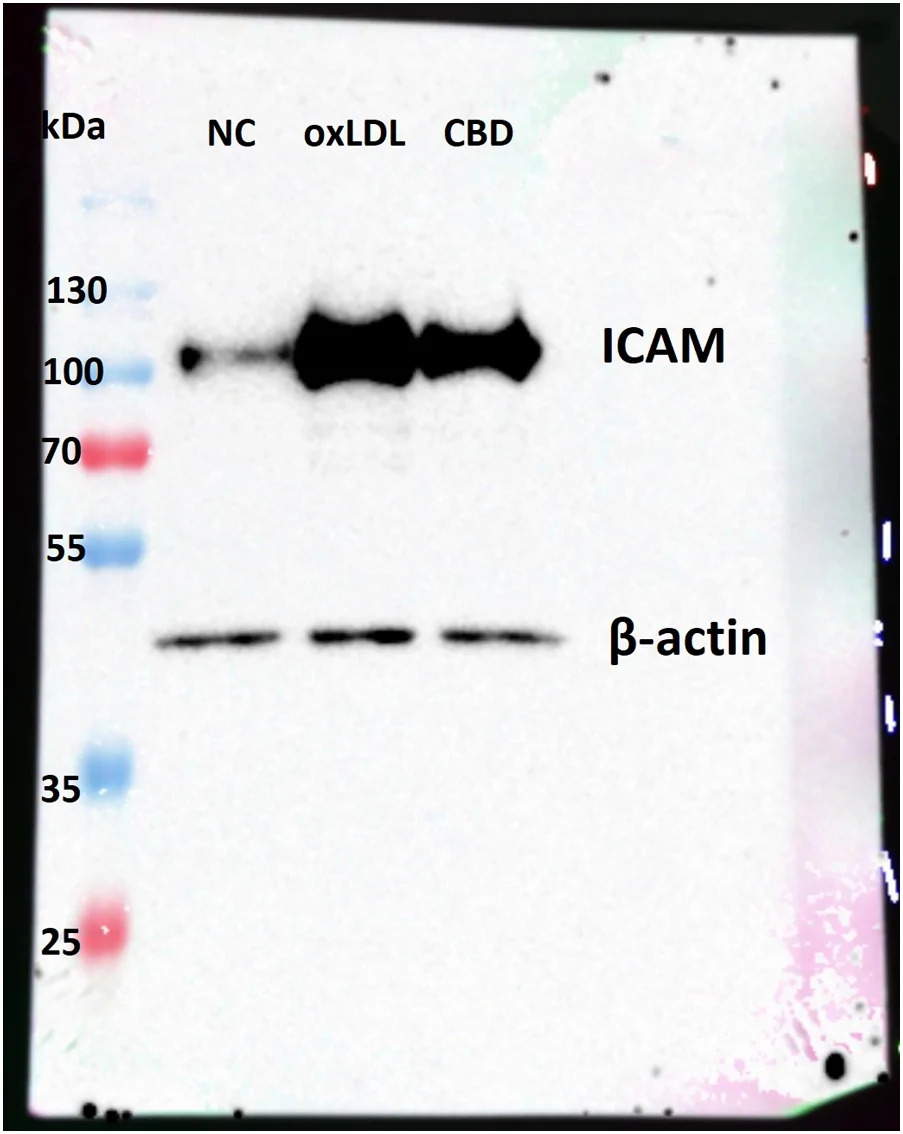
Complete Western blot corresponding to biological replicate 1 used for the quantification shown in Figure 6C. Human coronary artery endothelial cells (HCAEC) were treated with vehicle (NC), oxidized LDL (oxLDL), or oxLDL + cannabidiol (CBD, 2.5 mg/L). ICAM-1 and β-actin are shown together with the molecular weight marker.

**Supplemental Figure S6:**
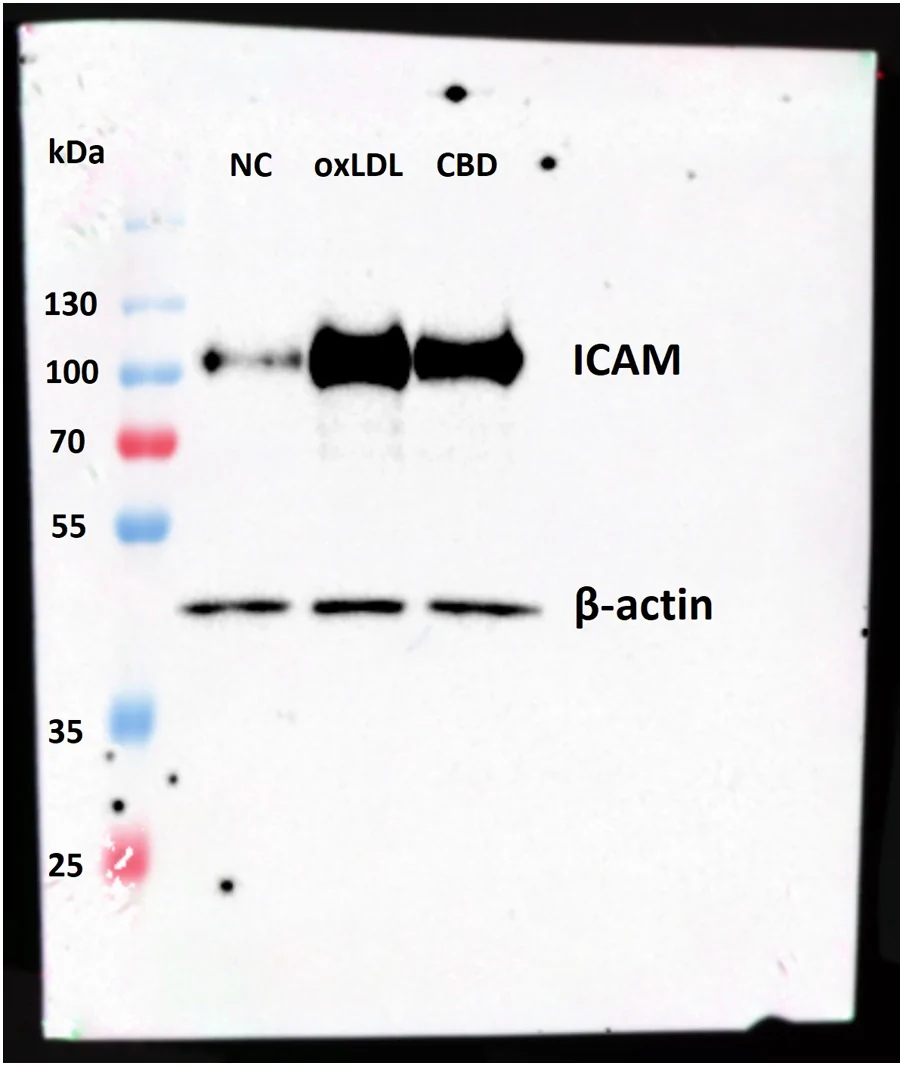
Complete Western blot corresponding to biological replicate 2 used for the quantification shown in Figure 6C. Human coronary artery endothelial cells (HCAEC) were treated with vehicle (NC), oxidized LDL (oxLDL), or oxLDL + cannabidiol (CBD, 2.5 mg/L). ICAM-1 and β-actin are shown together with the molecular weight marker.

**Supplemental Figure S7:**
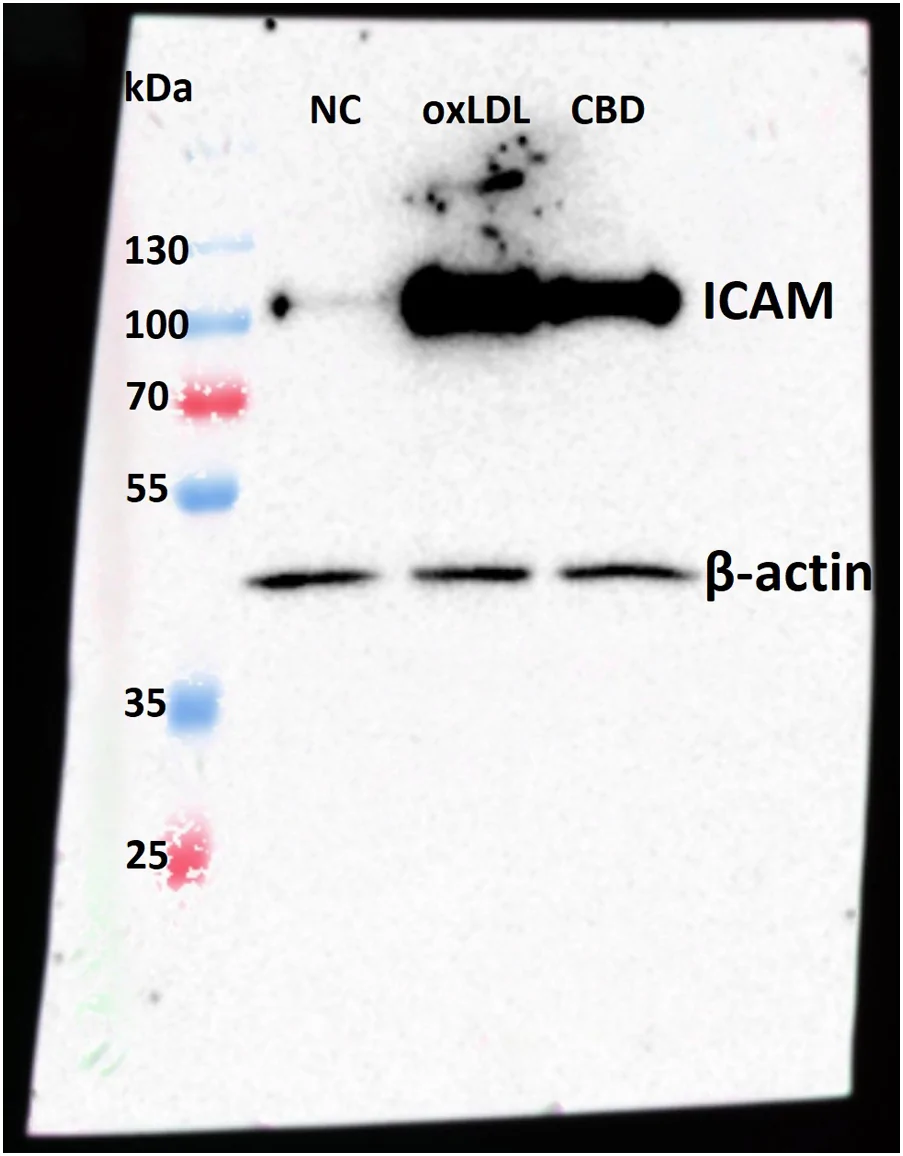
Complete Western blot corresponding to biological replicate 3 used for the quantification shown in Figure 6C. Human coronary artery endothelial cells (HCAEC) were treated with vehicle (NC), oxidized LDL (oxLDL), or oxLDL + cannabidiol (CBD, 2.5 mg/L). ICAM-1 and β-actin are shown together with the molecular weight marker.

To assess the direct effects of CBD on endothelial cells under pro-atherogenic conditions, we first determined non-cytotoxic concentrations suitable for subsequent in vitro experiments. After 72 h of exposure, CBD reduced human coronary artery endothelial cell (HCAEC) viability in a concentration-dependent manner, with a calculated IC_50_ of 4.0 ± 0.1 mg/L. Based on these results, a maximum concentration of 2.5 mg/L (corresponding to IC_20_) was selected for further experiments. To model early stages of atherosclerosis, HCAEC were exposed to oxidized low-density lipoproteins (oxLDL), which consistently induced oxidative stress and increased the production of inflammatory mediators. Under these conditions, CBD significantly reduced oxLDL-induced secretion of IL-6 and MCP-1 compared with oxLDL-treated cells (Fig. 6A, B). In parallel, Western blot analysis revealed reduced expression of the endothelial adhesion molecule ICAM in CBD-treated cells, indicating attenuation of endothelial activation (representative blot in Fig. 6C; complete blots are provided in Supplementary Figs. S5–S7). Consistent with these findings, measurements of cellular antioxidant activity demonstrated that CBD significantly decreased oxLDL-induced oxidative stress at 2, 4, and 24 h across all tested concentrations, suggesting a sustained antioxidant effect in endothelial cells (Fig. 6D).

### CBD induces a mild, male-specific increase in ALT without histological hepatotoxicity

Serum liver enzymes were evaluated by ART ANOVA with ART-C contrasts. CBD was associated with a mild, male-specific increase in serum ALT: both the main effect of treatment and the Treatment × Sex interaction were significant, with ALT rising in males (median 0.56–0.96 μkat/L) but unchanged in females, whereas the within-male contrast did not reach significance after correction. Serum AST did not differ by treatment in either sex (Supplementary Table S7). On histological examination, hepatocellular steatosis was present in all animals irrespective of treatment, predominantly diffuse and comprising a variable mixture of macro- and microvesicular components. Scattered foci of neutrophils with occasional single-cell hepatocyte dropout were observed and attributed to peri-mortem tissue handling rather than treatment. No features of toxic hepatocellular injury were detected. Together, these findings indicate no histological evidence of CBD-induced hepatotoxicity; the modest ALT elevation is consistent with the known association between CBD and transaminase levels and may in part reflect the background hepatic steatosis of this model. Given the small sample size, this signal warrants attention in larger studies.

## Discussion

Cannabidiol has demonstrated beneficial effects in various inflammatory and metabolic disease models. In the present study, using ApoE^−/−^ mice fed a Western-type diet as a model of atherosclerosis, we show that long-term administration of a CBD nanoemulsion reduces aortic plaque formation in a sex-dependent manner, with efficacy restricted to males. The mechanisms underlying this effect likely involve attenuation of mitochondrial oxidative metabolism, blunting of oxidative and inflammatory endothelial activation, accompanied by species-level changes in the circulating lipidome, including enrichment of ether-linked lipids.

### CBD and mitochondrial respiration

Oxidative stress is a well-known contributor to atherogenesis (24). Mitochondrial electron transport inherently generates reactive oxygen species (ROS), and under pathological conditions, excess mitochondrial ROS promotes mitochondrial dysfunction and vascular inflammation (25). CBD has been reported to modulate mitochondrial respiration and ROS production, although findings vary by model and concentration. CBD can suppress mitochondrial function in a manner associated with reduced cell viability or apoptosis in cancer cell lines (26, 27, 28) and neuron-like cells (29), whereas in stress-related contexts it exerts protective effects by limiting mitochondrial ROS generation and preserving cell viability (30, 31, 32, 33, 34, 35). Extracts from cannabis have also been shown to inhibit LDL oxidation and foam cell formation (36). These findings are further supported by our in vitro data, where CBD attenuated oxLDL-induced oxidative stress and inflammatory activation in endothelial cells, indicating a direct vascular effect under pro-atherogenic conditions.

Our proteomic findings demonstrate downregulation of mitochondrial pathways, including aerobic respiration, respiratory chain function, and complex I assembly, in CBD-treated males. These changes are consistent with reduced mitochondrial ROS production and provide a mechanistic link between CBD-induced decreases in oxidative metabolism and attenuated plaque formation.

### CBD and the serum lipid profile

Serum dyslipidemia is a major risk factor for atherosclerosis, typically addressed by interventions targeting circulating TAG and cholesterol levels. In our study, CBD did not alter standard serum lipid parameters (TAG, TC, HDL-C, LDL-C). Expected sex-dependent differences were observed, particularly lower TAG levels in females, but CBD had no lipid-lowering effect in either sex, contrasting with previous reports (37). Although HDL-C was higher in CBD-treated males than in females, the same pattern was present in untreated controls, indicating that these differences were sex-related rather than treatment-induced. It is worth noting that conventional lipid markers such as TAG, HDL-C, and others represent aggregate measurements and do not capture the molecular diversity within lipid classes.

In contrast, lipidomic profiling provides species resolution at the level of individual lipid species, without distinguishing their specific lipoprotein carriers, and allows detection of molecular lipid changes relevant to pathophysiology. Thus, clinical lipid markers and non-targeted lipidomics offer complementary but non-overlapping perspectives on lipid metabolism.

The serum lipidome changes are consistent with patterns typically induced by a Western-type diet rich in fat and cholesterol. High-fat feeding has been previously shown to elevate ceramides, hexosyl-ceramides, and sphingomyelins, while reducing selected lysophosphatidylcholines in mouse serum (38). Another study presents moderately but consistently increased levels of free cholesterol and cholesteryl esters for mice fed a Western diet when compared to controls, across various mice models (39). Such alterations reflect predominantly diet-driven remodeling of circulating lipid classes, together with further genotype and/or phenotype-dependent metabolic differences, and are partially confirmed with our results (see Supplementary Table S8, summarizing the serum lipidome of ApoE^−/−^ mice fed Western diet compared with C57BL/6J mice fed standard diet).

Consistent with this predominantly diet-driven lipidome, unsupervised analysis separated samples primarily by sex rather than CBD treatment, indicating that CBD did not globally remodel the circulating lipidome. Nevertheless, when focusing specifically on ApoE^−/−^ mice, the subtle lipidomic differences observed between CBD- and vehicle-treated animals, together with the pronounced sex-dependent variation, likely reflect the complexity of interacting metabolic pathways influenced by both CBD, a bioactive compound with pleiotropic biological effects (40, 41), and intrinsic sex-dependent metabolism (42, 43, 44). We therefore asked whether individual lipid species, rather than the lipidome as a whole, tracked plaque burden. In males, the species most strongly and negatively correlated with plaque area were dominated by ether-linked (plasmanyl and plasmenyl) triacylglycerols, which were also increased by CBD. Ether-TAGs belong to the broader family of ether lipids that includes plasmalogens, characterized by an ether bond at the sn-1 position of the glycerol backbone rather than the more common ester linkage. Plasmalogens are abundant membrane phospholipids with well-established antioxidant properties: their vinyl-ether bond acts as a sacrificial target for reactive oxygen species, limiting LDL oxidation (45). Moreover, experimental plasmalogen supplementation reduces plaque area and vascular inflammation in ApoE−/− mice, while plasmalogen remodeling has been implicated in macrophage responses to oxysterols and plaque vulnerability (46, 47). Ether-TAGs are neutral storage lipids proposed to share biosynthetic intermediates with ether-phospholipids and may serve as reservoirs for ether-linked fatty alcohols. Taken together, these findings suggest that a CBD-driven increase in ether lipids may represent a biologically plausible link to reduced oxidative burden. However, our data do not establish such a link. The inverse association between ether-TAG and plaque was nominal, and a class-level specificity screen showed that it was not specific to ether-TAG: composites of all detected lipid classes trended negatively with plaque, and none survived correction for multiple testing, with the ether-TAG composite ranking in the middle of this distribution. An exploratory mediation analysis of the CBD to ether-TAG to plaque path was not significant. We therefore interpret the CBD-induced ether-TAG enrichment as a biologically plausible, hypothesis-generating observation rather than a demonstrated mediator of plaque protection, and we anchor the mechanistic interpretation of our data on the convergent proteomic and in vitro evidence for attenuated oxidative burden. If confirmed in adequately powered studies, the selective enrichment of ether-linked triacylglycerols would nonetheless raise the possibility that the structural form of circulating triglycerides, rather than their total level, influences atherogenic risk (48, 49, 50), and would motivate targeted investigation of the interplay between ether-lipid synthesis, redox homeostasis, and cholesterol metabolism in CBD-mediated vascular protection.

### Sex-dependent effects of CBD

Growing evidence indicates that cannabinoid effects differ markedly between males and females, influenced by hormonal status, pharmacokinetics, and receptor expression patterns (51). Most prior studies have focused on neurobehavioral endpoints (52, 53, 54), with limited data on sex-specific metabolic or vascular responses. Preclinical studies on CBD in atherosclerosis have been conducted almost exclusively in males, leaving a significant gap in understanding female responses.

Our study provides the first direct comparison of CBD effects between sexes in an atherosclerosis model. We observed pronounced sex differences not only in plaque size—the primary endpoint—but also in the proteomic and lipidomic responses to CBD. Collectively, these findings underscore the necessity of incorporating sex as a biological variable in future preclinical and translational research on CBD and cardiovascular disease.

## Conclusion

The integrated analyses show that CBD modulates metabolic and oxidative pathways relevant to atherogenesis in a sex-dependent manner. Chronic CBD administration reduced aortic plaque burden exclusively in male ApoE−/− mice, despite no changes in body weight or conventional lipid parameters. Proteomic profiling revealed a coordinated downregulation of mitochondrial oxidative and stress-related pathways, and in vitro CBD attenuated oxLDL-induced oxidative stress and inflammatory activation in endothelial cells, together pointing to a reduction in vascular oxidative burden as a plausible mechanism. In parallel, CBD produced subtle, species-level changes in the serum lipidome rather than a global shift, including enrichment of ether-linked triacylglycerols connected to antioxidant plasmalogen metabolism; whether this contributes causally to plaque protection remains an open question for future work. Together, these findings identify sex-specific, oxidative and metabolic responses as candidate determinants of CBD's anti-atherogenic effect and motivate targeted studies of ether-lipid flux, lipoprotein-specific signatures, and mitochondrial adaptation in both sexes.

## Data availability

The data will be provided upon reasonable request.

## Supplemental data

This article contains supplemental data.

## Conflict of interest

The authors declare that they have no conflicts of interest with the contents of this article.

## Supplemental data

Supplemental Table S1 Plaque area: descriptive statistics, robust/rank triangulation, and sensitivity.

Supplemental Table S2 Aortic root proteome, list of differentially abundant proteins (SAM FDR < 0.1).

Supplemental Table S3 Effect of CBD treatment and sex on routine serum lipids (aligned rank transform ANOVA and within-sex ART-C contrasts).

Supplemental Table S4 Correlation of lipid species with atherosclerotic plaque area in male mice and differential abundance between vehicle- and CBD-treated groups. Pearson and Spearman correlation coefficients (with corresponding p- and Benjamini–Hochberg-adjusted q-values) are shown together with group geometric means, fold changes, and results of differential abundance testing (Mann–Whitney, Welch's *t* test, and limma moderated *t* test).

Supplemental Table S5 Correlation of lipid species with atherosclerotic plaque area in female mice and differential abundance between vehicle- and CBD-treated groups. Pearson and Spearman correlation coefficients (with corresponding p- and Benjamini–Hochberg-adjusted q-values) are shown together with group geometric means, fold changes, and results of differential abundance testing (Mann–Whitney, Welch's *t* test, and limma moderated *t* test).

Supplemental Table S6 Class-level specificity screen (whole class, treatment-blind).

Supplemental Table S7 Effect of CBD treatment and sex on liver transaminases (aligned rank transform ANOVA and within-sex ART-C contrasts).

Supplemental Table S8 Comparison of serum lipidome of ApoE^−/−^ mice fed a Western diet and of C57BL/6J mice fed a standard diet.

Supplemental Figure S1 Body weight of ApoE^−/−^ mice during 12 weeks of high-fat diet feeding with or without CBD treatment. Data are presented as mean ± SEM (n = 8 per group).

Supplemental Figure S2 Aortic plaque area in CBD- and vehicle-treated ApoE^−/−^ mice. Representative unprocessed images of stained aortas (with pins). F, female; M, male.

Supplemental Figure S3 *Differential abundance analysis of aortic proteins between CBD- and vehicle-treated male mice*, estimated using (A) Reproducibility-Optimized Test Statistic (ROTS) and (B) Significance Analysis of Microarrays (SAM). Red dots indicate proteins with statistically significant differences in abundance (FDR < 0.1).

Supplemental Figure S4 Representative MS/MS spectra of the three plasmanyl-TAG species correlating with plaque area.
