# Supplementary material for: Cannabidiol attenuates atherosclerosis in male ApoE−/− mice with sex-dependent lipidomic and proteomic remodeling
Source: J Lipid Res. 2026 Aug 10;67(9):101120. doi: 10.1016/j.jlr.2026.101120 (PMC13586724; doi:10.1016/j.jlr.2026.101120)
Supplement: Supplementary file 1 [file mmc10.pdf]

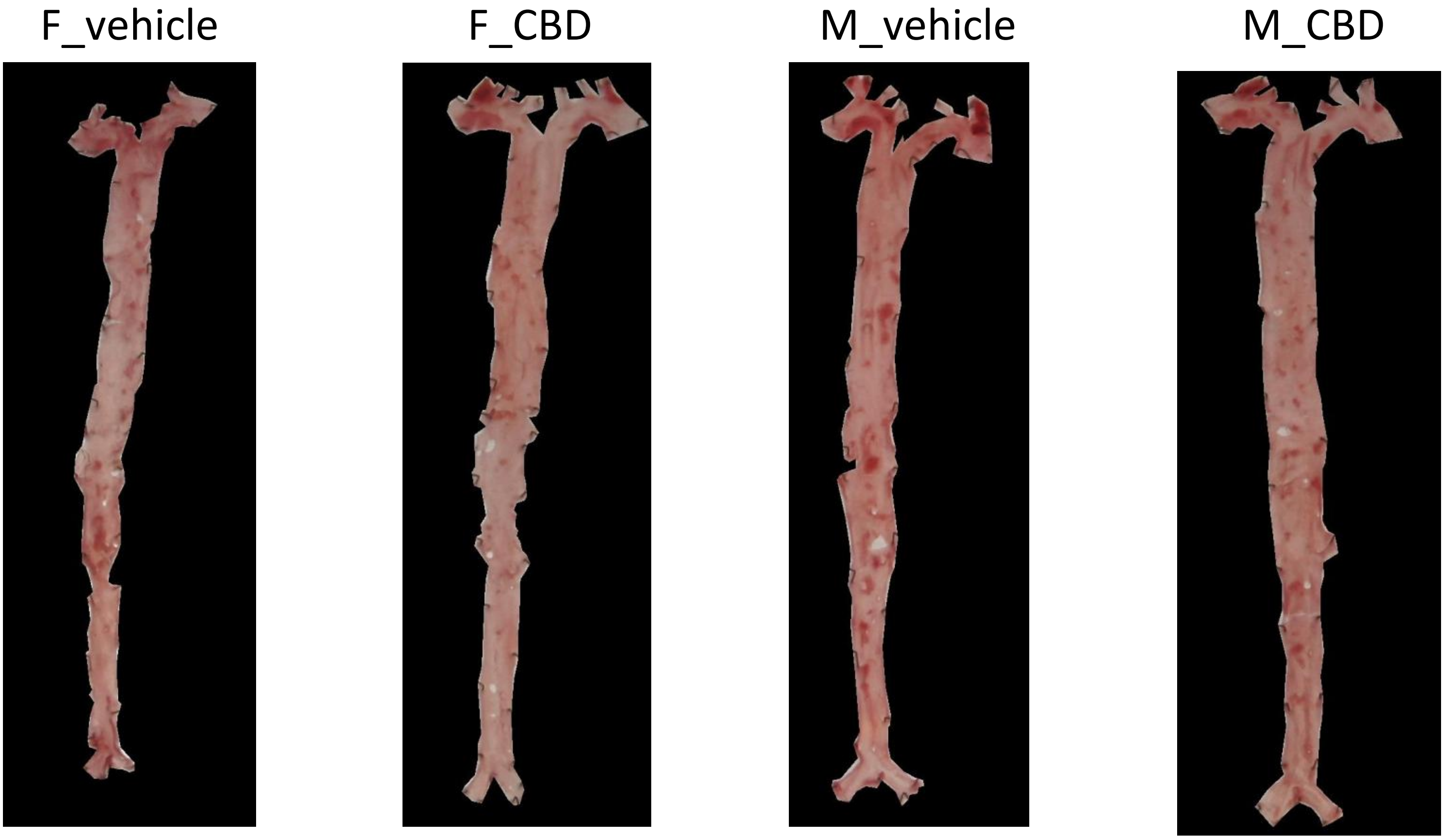

Supplementary Figure 2 *Aortic plaque area in CBD- and vehicle-treated $ApoE^{-/-}$ mice.* Representative unprocessed images of stained aortas (with pins). F, female; M, male.
