# Supplementary material for: Cannabidiol attenuates atherosclerosis in male ApoE−/− mice with sex-dependent lipidomic and proteomic remodeling
Source: J Lipid Res. 2026 Aug 10;67(9):101120. doi: 10.1016/j.jlr.2026.101120 (PMC13586724; doi:10.1016/j.jlr.2026.101120)
Supplement: Supplementary file 2 [file mmc11.pdf]

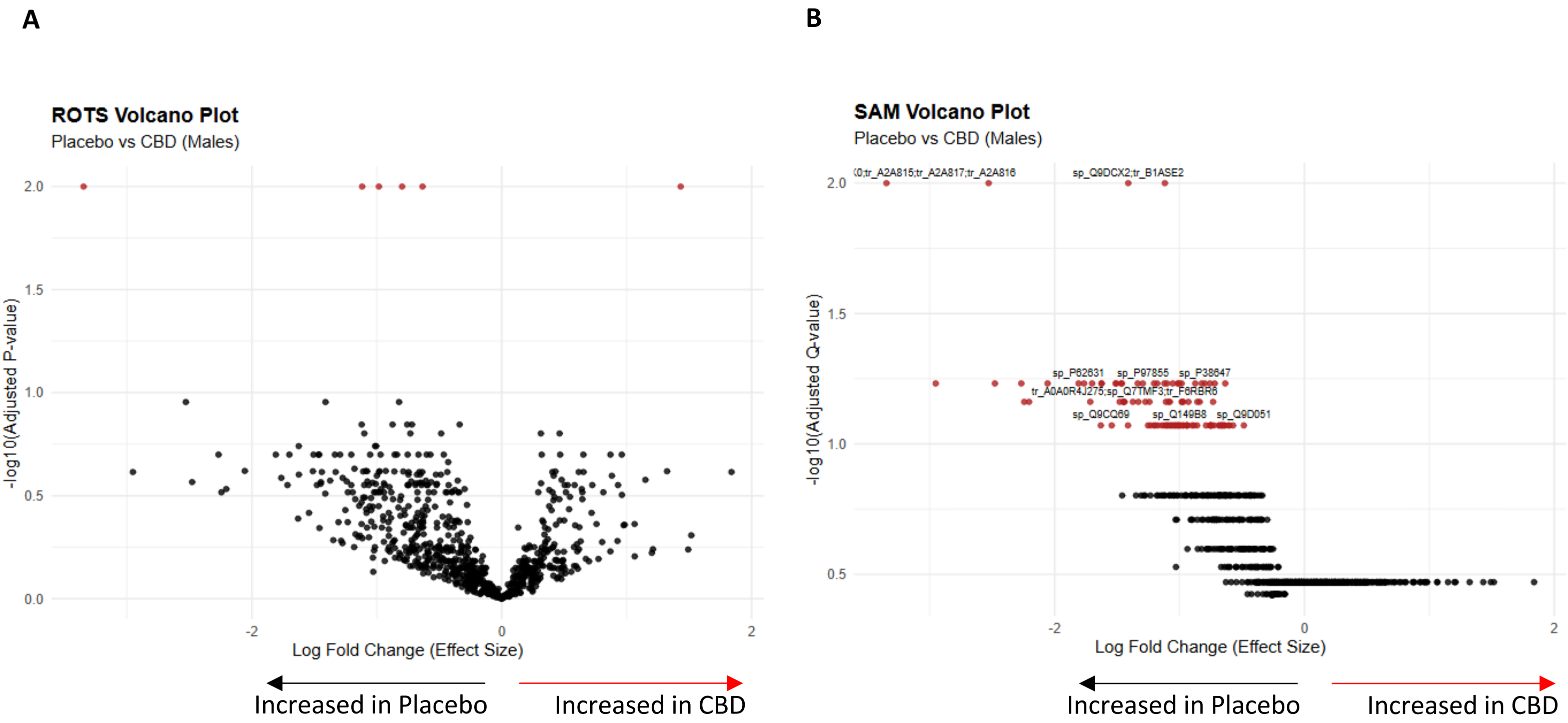

Supplementary Figure 3 *Differential abundance analysis of aortic proteins between CBD- and vehicle-treated male mice*, estimated using (A) Reproducibility-Optimized Test Statistic (ROTS) and (B) Significance Analysis of Microarrays (SAM). Red dots indicate proteins with statistically significant differences in abundance (FDR < 0.1).
