# Supplementary material for: Cannabidiol attenuates atherosclerosis in male ApoE−/− mice with sex-dependent lipidomic and proteomic remodeling
Source: J Lipid Res. 2026 Aug 10;67(9):101120. doi: 10.1016/j.jlr.2026.101120 (PMC13586724; doi:10.1016/j.jlr.2026.101120)
Supplement: Supplementary file 3 [file mmc12.pdf]

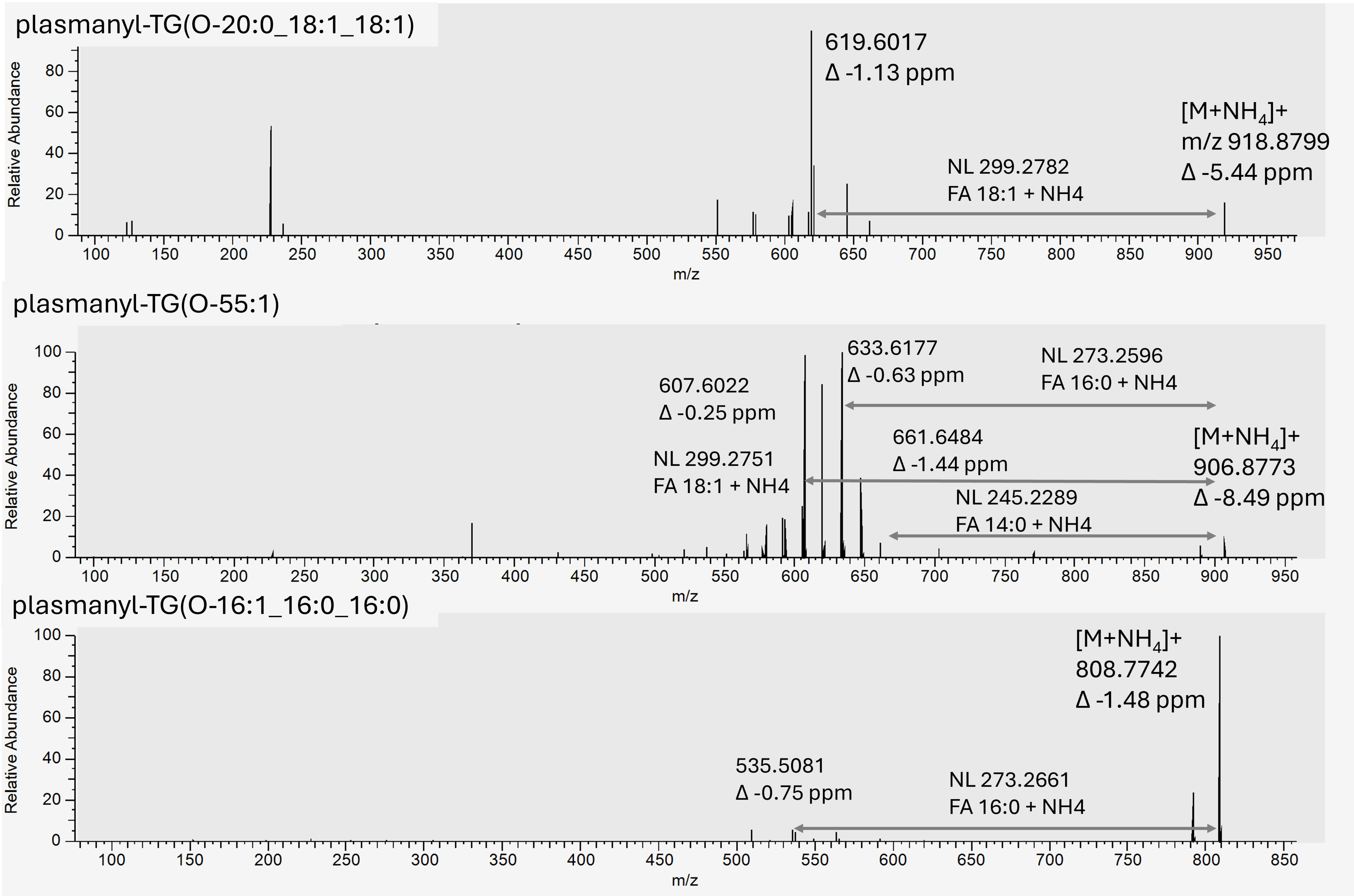

plasmanyl-TG(O-20:0_18:1_18:1)
Relative Abundance
619.6017
Δ -1.13 ppm
[M+NH4]+
m/z 918.8799
Δ -5.44 ppm
NL 299.2782
FA 18:1 + NH4
m/z
plasmanyl-TG(O-55:1)
633.6177
Δ -0.63 ppm
NL 273.2596
FA 16:0 + NH4
607.6022
Δ -0.25 ppm
661.6484
Δ -1.44 ppm
[M+NH4]+
906.8773
Δ -8.49 ppm
NL 299.2751
FA 18:1 + NH4
NL 245.2289
FA 14:0 + NH4
plasmanyl-TG(O-16:1_16:0_16:0)
[M+NH4]+
808.7742
Δ -1.48 ppm
535.5081
Δ -0.75 ppm
NL 273.2661
FA 16:0 + NH4
