# Supplementary material for: Cannabidiol attenuates atherosclerosis in male ApoE−/− mice with sex-dependent lipidomic and proteomic remodeling
Source: J Lipid Res. 2026 Aug 10;67(9):101120. doi: 10.1016/j.jlr.2026.101120 (PMC13586724; doi:10.1016/j.jlr.2026.101120)
Supplement: Supplementary file 4 [file mmc9.pdf]

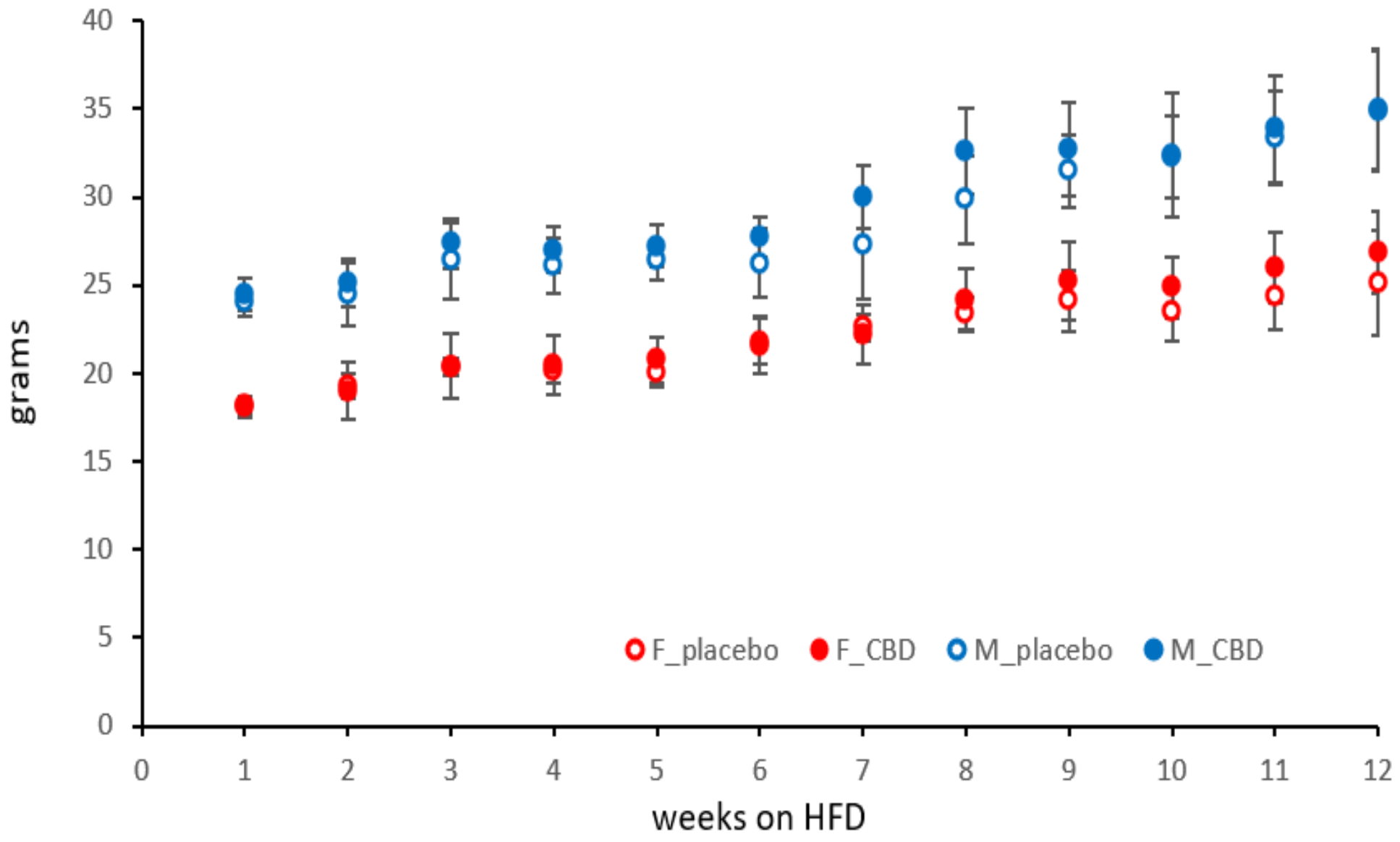

Supplementary Figure 1: *Body weight of ApoE⁻/⁻ mice during 12 weeks of high-fat diet feeding with or without CBD treatment. Data are presented as mean ± SEM (n = 8 per group).*
