# Supplementary material for: Cannabidiol attenuates atherosclerosis in male ApoE−/− mice with sex-dependent lipidomic and proteomic remodeling
Source: J Lipid Res. 2026 Aug 10;67(9):101120. doi: 10.1016/j.jlr.2026.101120 (PMC13586724; doi:10.1016/j.jlr.2026.101120)
Supplement: Supplementary file 5 [file mmc8.docx]

**Supplementary Table S8:** *Comparison of serum lipidome of ApoE⁻/⁻ mice fed a Western diet and of C57BL/6J mice fed a standard diet*

| **Lipid class** | **Number of lipid species** | **Average FC female** | **Average FC male** |
| --- | --- | --- | --- |
| AcylCarnitines | 4 | 5.15 | 6.02 |
| Cholesteryl esters | 11 | 8.94 | 7.55 |
| Ceramides | 34 | 4.90 | 4.43 |
| DAG | 5 | 25.93 | 12.69 |
| FAHFAs | 8 | 4.32 | 3.73 |
| Hexosyl ceramides | 16 | 5.93 | 4.91 |
| Lysophospholipids (LPC+LPE) | 22 | 7.10 | 5.76 |
| PC | 78 | 8.01 | 7.26 |
| PE | 3 | 7.17 | 4.60 |
| PI | 10 | 9.91 | 8.18 |
| Sphingomyelins | 7 | 4.48 | 3.91 |
| TAG | 52 | 6.45 | 7.26 |
| plasmenyl-TAG | 11 | 0.05 | 0.03 |

Fold Change > 1 means lipid classes increased (in average) in mice fed with Western diet, Fold Change < 1 means lipid classes decreased (in average) in mice fed with Western diet.
